# Executive function and relation to static balance metrics in chronic mild TBI: A LIMBIC-CENC secondary analysis

**DOI:** 10.3389/fneur.2022.906661

**Published:** 2023-01-11

**Authors:** Susanne M. van der Veen, Robert A. Perera, Laura Manning-Franke, Amma A. Agyemang, Karen Skop, Scott R. Sponheim, Elisabeth A. Wilde, Alexander Stamenkovic, James S. Thomas, William C. Walker

**Affiliations:** ^1^Department of Physical Therapy, College of Health Professions, Virginia Commonwealth University, Richmond, VA, United States; ^2^Department of Physical Medicine and Rehabilitation, School of Medicine, Virginia Commonwealth University, Richmond, VA, United States; ^3^Department of Biostatistics, Virginia Commonwealth University, Richmond, VA, United States; ^4^Department of Physical Medicine and Rehabilitation Services, James A. Haley Veterans' Hospital, Tampa, FL, United States; ^5^Minneapolis VA Health Care System, Veterans Affairs Medical Center, Minneapolis, MN, United States; ^6^Department of Psychiatry and Behavioral Sciences, University of Minnesota, Minneapolis, MN, United States; ^7^Department of Physical Medicine and Rehabilitation, Michael E. DeBakey VA Medical Center, Houston, TX, United States; ^8^Physical Medicine and Rehabilitation, Baylor College of Medicine, Houston, TX, United States; ^9^Department of Neurology, University of Utah, Salt Lake City, UT, United States; ^10^Richmond Veterans Affairs (VA) Medical Center, Central Virginia VA Health Care System, Richmond, VA, United States

**Keywords:** balance, executive function, traumatic brain injury, concussion, gait, cognition, military

## Abstract

**Introduction:**

Among patients with traumatic brain injury (TBI), postural instability often persists chronically with negative consequences such as higher fall risk. One explanation may be reduced executive function (EF) required to effectively process, interpret and combine, sensory information. In other populations, a decline in higher cognitive functions are associated with a decline in walking and balance skills. Considering the link between EF decline and reduction in functional capacity, we investigated whether specific tests of executive function could predict balance function in a cohort of individuals with a history of chronic mild TBI (mTBI) and compared to individuals with a negative history of mTBI.

**Methods:**

Secondary analysis was performed on the local LIMBIC-CENC cohort (*N* = 338, 259 mTBI, mean 45 ± STD 10 age). Static balance was assessed with the sensory organization test (SOT). Hierarchical regression was used for each EF test outcome using the following blocks: (1) the number of TBIs sustained, age, and sex; (2) the separate Trail making test (TMT); (3) anti-saccade eye tracking items (error, latency, and accuracy); (4) Oddball distractor stimulus P300 and N200 at PZ and FZ response; and (5) Oddball target stimulus P300 and N200 at PZ and FZ response.

**Results:**

The full model with all predictors accounted for between 15.2% and 21.5% of the variability in the balance measures. The number of TBI's) showed a negative association with the SOT2 score (*p* = 0.002). Additionally, longer times to complete TMT part B were shown to be related to a worse SOT1 score (*p* = 0.038). EEG distractors had the most influence on the SOT3 score (*p* = 0.019). Lastly, the SOT-composite and SOT5 scores were shown to be associated with longer inhibition latencies and errors (anti-saccade latency and error, *p* = 0.026 and *p* = 0.043 respectively).

**Conclusions:**

These findings show that integration and re-weighting of sensory input when vision is occluded or corrupted is most related to EF. This indicates that combat-exposed Veterans and Service Members have greater problems when they need to differentiate between cues when vision is not a reliable input. In sum, these findings suggest that EF could be important for interpreting sensory information to identify balance challenges in chronic mTBI.

## 1. Introduction

Cognitive decline can occur due to natural aging and/or as a result of numerous pathological mechanisms (traumatic brain injury, stroke, psychosis, etc). The resulting cognitive decline and limited executive function (EF) have been related to the risk of falling and control of gait. Elderly and stroke survivors have been known to lag or slow down when walking to audio or visual cues (1, 2), be less accurate at dual tasks (auditory Stroop-task) during walking (3), and even stop walking when talking (4, 5). Patients with traumatic brain injury (TBI) can suffer symptoms of dizziness, nausea, and postural instability that persist for over 3 months in 40%−50% of mild TBI patients (6–11) and longer when caused by blast exposure (e.g., military injury and industrial accidents). In combination with these balance impairments, patients with TBI often present with altered EF (12–14). As the ability to remain balanced may be dictated by the processing, interpretation, and combining of sensory information, investigation of the relationship between postural balance and EF in a TBI cohort may shed light on cognitive–sensory mechanisms driving balance dysfunction. Additionally, the amplitude of event-related potentials (ERP) reflective of attention, conflict monitoring, and inhibition to visual and auditory stimuli during oddball tasks may be related to measures of walking speed integration of sensory information (i.e., vestibular, visual, and somatosensory signals) for maintaining balance.

Balance depends on the input and integration of signals from visual, vestibular, and somatosensory systems. The integration of information in these systems provides movement cues and reweighting to minimize sway and optimize postural stability and movement efficiency. For example, patients with vestibular loss become more reliant on proprioceptive and visual sensory information for balance control (15). Further loss of vision, such as standing in a dark room, elicits a dominant role of proprioceptive information for balance control. This is especially true during challenging mobility tasks, such as walking in cluttered terrain, which are likely to involve higher-order cognitive processes that regulate abilities such as working memory, alternating and selective attention, inhibitory control, cognitive flexibility, problem-solving, organizational skills, and abstract reasoning.

Quantifying EF during tasks that involve maintaining balance and household or community ambulation is particularly challenging. In general, the assessments of executive functioning take the form of self-report scales, Stroop-tasks, reaction time tasks, and dual tasks that often examine different components of the cognitive-motor process. Specific examples, including the oddball and anti-saccade tests, reflect conflict monitoring and de-programming/inhibition abilities (16, 17) that are measured through the P300 amplitude responses from ERPs recorded through electroencephalogram (EEG). P300 amplitudes decrease during dual tasks, indicating that the amplitude of the P300 response is an indication of resource availability. Furthermore, P300 amplitudes from oddball responses were reduced during walking with a rucksack compared to sitting and interpreted as a prioritization of cortical processes needed while walking compared to doing the cognitive task alone [i.e., oddball task (18)]. Several EEG studies during full-body movement have been published with improvements in methodology and techniques for movement artifact reduction, thus allowing for greater interpretation of higher cognitive function during gait and gait adaptations (18–21). Faster walking showed reduced spectral alpha and beta power in the sensorimotor cortex, indicating greater cortical involvement likely necessary for faster processing of sensory feedback (19). This activity is time synchronized to spectral power increases in the supplementary motor area, premotor cortex, and posterior parietal cortex that occur with step adjustments, suggesting the involvement of these brain regions in gait adaptations necessary to interrupt subcortical control of gait cycle parameters. Specifically, the posterior parietal cortex tracks obstacle location for planning foot placement nearly two steps ahead of reaching the obstacle requiring step adjustment (21). This process is likely to rely heavily on the interpretation of sensory input and the integration of this information. However, methods used to assess what brain regions are used during whole-body movement are complicated, and analyses are greatly affected by levels of noise induced by whole-body movement and equipment used to design the experimental setup.

Some studies have attempted to generate models for gait and balance and determine which gait and balance characteristics best relate to cognition (22). For example, in patients with Parkinson's disease (23), balance measures (e.g., the center of pressure sway area/jerkiness, sway velocity, and sway frequency in both the sagittal and frontal plane) account for 84.5% of the variance in changes in cognitive measures that tap attention and visuospatial abilities. Additionally, Morris et al. found gait measures, including pace/turning, and gait variability (stride length, foot strike angle, gait cycle duration, stance time, and swing time), have been strongly associated with attention and EF (23).

As the ability to maintain postural balance may be dictated by the ability to process, interpret, and combine sensory information, we assessed the relationship between cognitive functions as measured by EEG ERP (P300 and N200) amplitudes to postural stability as measured through the computerized sensory organization test (SOT). We hypothesized that the individuals with TBI would present with decreased postural stability when sensory inputs to the central nervous system are manipulated and would have more limited EF as indicated by smaller differences between EEG amplitudes (P300) for target and distractor.

## 2. Methods

### 2.1. Design

The study utilized an observational design with cross-sectional analyses using hierarchical regression to examine the predictive value of EF measures, including ERP N200 and P300, anti-saccade, and trail-making tests on gait and balance.

### 2.2. Setting

This study reports findings of an interim analysis from the Long-Term Impact of Military Relevant Brain Injury Consortium/Chronic Effects of Neurotrauma Consortium (LIMBIC-CENC) prospective longitudinal study (PLS) of the late effects of military combat deployment. For more information on the overarching study's background, breadth, and overall objectives, refer to the prior publication by Walker et al. (24).

### 2.3. Participants

Participants were recruited primarily from mass letter mailing campaigns to registered patients at Richmond Veterans Affairs Medical Center (VAMC) and secondarily by advertisements, flyers, community outreach, and clinician referrals. The intended population for the overarching observational study is service members and veterans who experienced combat situation(s) and have a history of a varying number, from none to many, of prior mild TBI (mTBI). The only exclusion criteria were (1) a history of moderate or severe TBI as defined by either (a) initial Glasgow Coma Scale <13, (b) coma duration of >0.5 h, (c) post-traumatic amnesia (PTA) duration of >24 h, or (d) traumatic intracranial lesion on head computerized tomography; or (2) history of (a) major neurological disorder (e.g., stroke, spinal cord injury), (b) major psychiatric disorder (e.g., schizophrenia) with major defined as resulting in a significant decrement in functional status or loss of independent living capacity. Notably, PTSD and mood disorders were not considered exclusionary. The intended sample for these analyses are all participants enrolled and completing enrollment assessments before this dataset was extracted at the site where EEG was conducted (VAMC) and who were physically able to complete the SOT protocol (*n* = 338).

### 2.4. Assessments and measures overview

The full breadth of assessments and data collection measures used in the overarching study are described elsewhere (24). For these analyses, the primary dependent variables are balance, gait, and EF measures described below.

#### 2.4.1. Potential concussive event identification and TBI diagnosis

The in-depth structured interview process in this study entailed screening for all potential concussive events (PCEs) during military deployments and across the entire lifetime, including childhood, using a modification of the Ohio State University TBI Identification (OSU TBI-ID) instrument (25). Each PCE identified was interrogated to determine whether or not it was a true clinical mTBI *via* a detailed structured interview, the Virginia Commonwealth University retrospective Concussion Diagnostic Interview (VCU rCDI) (26). Each VCU rCDI rendered a preliminary TBI diagnosis of either mTBI with PTA, mTBI without PTA, or not mTBI through an embedded algorithm using the structured interview data and based on the DoD/VA common definition of mTBI. Algorithm-based TBI diagnosis was reviewed and vetted against the unstructured free-text portion of the interview and against any medical documents recorded in proximity to the event (i.e., first responder, emergency department, or in-theater documentation). Using this process, the site-principal investigator confirmed or overrode every preliminary algorithm mTBI diagnosis to yield the final diagnosis. The event was also assessed for TBI severity to ensure eligibility (any severity greater than mild was excluded from this study). If any doubt remained on the diagnosis of TBI, the event was adjudicated by a central diagnosis committee consisting of national experts in TBI. Further details on PCE and TBI identification can be found in a previous publication on this dataset (24).

**Figure 1:**
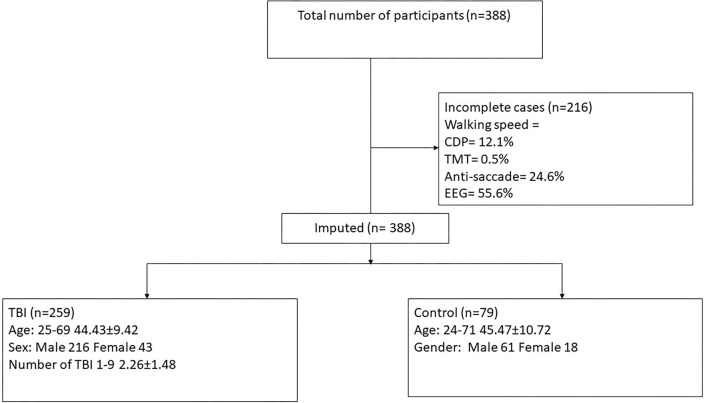
Consort diagram demonstrating participant selection for the current study. SOT, computerized dynamic posturography; TMT, trail-making test; EEG, auditory oddball task, measuring onset and amplitude of N200 and P300 responses at PZ and FZ; TBI, traumatic brain injury.

The lifetime mTBI diagnostic process described above yielded the number of lifetime mTBIs for each participant, from none to many. We also categorized participants into two main mTBI groups, positive (*n* = 259) and negative (*n* = 79) history (see Figure 1).

#### 2.4.2. Age

Age in years at the time of the evaluation.

#### 2.4.3. Gender

Self-identified gender was collected from each participant using a demographic questionnaire. This item was collected as queried in the CDC Behavioral Risk Factor Surveillance System (BRFSS), the nation's premier system of health-related surveys that collect data about health-related risk behaviors and chronic health conditions of the residents of the US (27).

#### 2.4.4. Balance

Postural stability was measured using the SOT protocol on the NeuroCom Smart Balance Master (NeuroCom; NeuroCom International, Inc., Clackamas, OR). Using a dual-plate force platform, the SOT generates equilibrium scores that compare the largest anterior–posterior movements of the subject over the trial to a theoretical limit for six sensory condition tasks. The sensory conditions were as follows: (1) eyes open with fixed surface and visual surroundings; (2) eyes closed with a fixed surface; (3) eyes open with a fixed surface and sway-referenced visual surroundings; (4) eyes open with a sway-referenced surface and fixed visual field; (5) eyes closed with a sway-referenced surface; and (6) eyes open with a sway-referenced surface and visual surroundings. Evaluators were trained and certified by an expert vestibular physical therapist; certification entailed assessing the videotape of the evaluator performing the SOT on a staff volunteer with further corrective training as needed until the performance was deemed satisfactory. Each subject performed three trials on the NeuroCom Smart Balance Master (for each of the six sensory conditions, resulting in 18 equilibrium trial scores, ranging from 0 (touching a support surface, shifting feet, or falling) to 100 (little or no sway). Average equilibrium scores were generated for each of the six conditions by averaging the three trial scores. The overall composite equilibrium score was calculated as a weighted average of these six scores (conditions 1 and 2 are weighted 1/3 as much as conditions 3 through 6).

As described by Cevette et al. (28), SOT equilibrium score profiles were considered non-credible if the average scores on conditions 1, 2, or 3 (easier conditions) were lower than on conditions 5 or 6 (more challenging conditions); this was true for a single participant (without mTBI, status 3 months after arthroscopic surgery of the right knee) whose SOT scores were treated as missing data.

#### 2.4.5. Executive functions

Three different assessments were used to measure cognitive and EF, all measuring slightly different aspects of the cognitive processes involving decision-making, attention, and processing speed. Each measure is explained more broadly below.

##### 2.4.5.1. Trail-making test

The trail-making test (TMT) is a test of visual attention and task switching (29). The TMT requires visuomotor integration while engaging in concurrent mental manipulation of numbers and letters, thus providing a measure of executive control. It can provide information about visual search speed, scanning, processing speed, mental flexibility, and executive functioning. It is also sensitive to detecting several cognitive impairments, such as Alzheimer's disease and dementia. Part A is a good measure of processing speed. Part B is generally quite sensitive to executive functioning since the test requires task switching (30).

##### 2.4.5.2. Electroencephalographic measures

Event-related potentials were used to probe neural coordination during multiple cognitive processes in the auditory domains. The testing paradigm measured well-studied ERPs, including the auditory early cortical potentials and P300 and N200 (oddball task consisting of non-target, target, and distractor stimuli) (31). The responses to the oddball task at the midline (PZ and FZ) to distractor and target stimulus were used as an indication of response selection, conflict monitoring, and inhibition.

##### 2.4.5.3. Abnormalities in eye movements (saccades and smooth pursuits)

Tracking of eye position in response to specific visual target movements was done with the EyeLink II, a non-invasive head-mounted eye-tracking human–machine interface system (32). In a recent pilot study, several CENC investigators showed differences between service members with chronic mTBI and unexposed participants using this system (32). An 8-min EyeLink II protocol was devised with 10 different stimulus tests, including challenging sequences, to minimize false-positive findings due to fatigue/boredom in under-challenged individuals. For these analyses, we considered only the anti-saccades test (performing the opposite movement of the stimulus relative to a vertical center line) used to depict conflict monitoring and inhibition (33). More specifically, the anti-saccade test is an eye movement task using three blocks of trials in which subjects look at a fixation point in the center of a computer screen and move their eyes upon the presentation of a laterally presented stimulus. In the first block (pro-saccade), subjects are instructed to move their eyes in the direction of the presented stimulus. In the second and third blocks (anti-saccade), subjects are instructed to move their eyes in the opposite direction of the presented stimulus and so must suppress the reflexive urge to look at a visual target. Latency (time from target movement to subject's primary response, computed as the difference between the time of target movement and time of peak velocity in primary saccade), error (the count of the number of eye movements where the sign of the velocity matches the sign of the target velocity), and accuracy (ratio of eye movement amplitude to target movement amplitude) are calculated using custom software (Matlab R2021a, The Mathworks, Natick, MA) and averaged over the two blocks of anti-saccade tests and over left and right eyes.

### 2.5. Data analysis

Participant characteristics were summarized using means and standard deviations or frequencies and percentages (see Table 1). Missing data were accounted for using the five-monotone method multiple imputations (the auto imputation in IBM statistics, SPSS 27). Five imputed datasets were created using a full condition specification (see Figure 1). The estimates were then combined, and standard errors were adjusted to account for the uncertainty due to missing data. Hierarchical regressions were performed on all participant data for each balance measure as independent variables with sensory tests grouped in the following sequential steps: (1) the number of TBIs sustained, age, and sex; (2) the separate TMT items; (3) separate eye-tracking items; (4) distractor P300 and N200 at PZ and FZ; and (5) target P300 and N200 at PZ and FZ. Separate regression analyses were carried out for each of the balance assessments (i.e., different scores on the SOT). Sensory measures were removed from the regression equations when multicollinearity was found [variance inflation factor (VIF) > 10]. Statistical significance was determined using a Benjamini–Hochberg correction for multiple comparisons (34), and reported *p*-values are before correction for multiple comparisons.

**Table 1:** Participant demographics, mean ± standard deviation, except for gender (male/female).

	**mTBI**		**Negative history of mTBI**		**Total**	
	**Mean**	**SD**	**Mean**	**SD**	**Mean**	**SD**
Age (mean/SD)	44.43	9.42	45.47	10.72	44.67	9.73
Sex (male/female)	216/43	–	61/18	–	277/61	–
#TBI's	2.26	1.481	0	0	1.73	1.61
Walking speed (m/s)	1.26	0.22	1.28	0.2	1.27	0.21
SOT composite	72.65	12.04	74.33	7.79	73.04	11.21
SOT1	91.16	5.42	91.19	5.1	91.17	5.34
SOT2	86.92	7.61	88.84	4.6	87.37	7.07
SOT3	86.83	8.78	88.78	5.19	87.28	8.13
SOT4	72.79	15.96	74.75	11.1	73.25	14.98
SOT5	59.59	16.77	61.49	13	60.03	15.97
SOT6	60.12	19.8	62.13	15.52	60.59	18.89

Additionally, we estimated the proportion of variance explained in the balance measures by the EF measures and the proportion of variance in the EF measures by the balance measures. This strategy is appropriate in this situation where a large set of measures are obtained to represent an individual's EF and balance; however, the focus is on the relationship between each set of measures rather than each measure individually.

## 3. Results

### 3.1. Participants

The study included data from 338 participants. All participants were included in analyses due to the use of multiple imputations. Of these 338 participants, 259 sustained at least one prior mTBI and 79 had a negative history of TBI. Those with positive TBI histories were a median of 9 years since their last mTBI. Table 1 shows the demographics of the participants.

### 3.2. SOT composite

Table 2 presents the complete hierarchical regression results for the SOT-composite score. Step 1 accounted for 5.0% of the variance in the SOT-composite score and revealed that none of the demographic measures showed sufficient evidence of an association with the SOT-composite score. Step 2 added TMT scores to the model, increased the variance accounted for 10.2%, and revealed that none of the TMT measures showed sufficient evidence of an association with the SOT-composite score. Step 3 added anti-saccade measures, increased the variance accounted for 11.7%, and showed insufficient evidence to conclude an association with the SOT-composite score. Step 4 added distractor EEG measures and increased variance accounted for 15.0%. The Step 4 model anti-saccade latency measure showed a statistically significant (*p* = 0.039) positive relationship with the SOT-composite score, indicating longer latencies are associated with better balance scores. Step 5 added EEG target measures and increased variance accounted for 17.5%, but again, none of the measures showed sufficient evidence of an association with the SOT-composite score.

**Table 2:** Results from the hierarchical regression for SOT composite, averaged over the six SOT balance assessments.

**SOT-composition**	**Model 1**				**Model 2**				**Model 3**				**Model 4**				**Model 5**			
	***B***	**SE**	***t***	***p*-Value**	***B***	**SE**	***t***	***p*-Value**	***B***	**SE**	***t***	***p*-Value**	***B***	**SE**	***t***	***p*-Value**	***B***	**SE**	***t***	***p*-Value**
*R* ^2^	0.05				0.102				0.117				0.15				0.175			
(Constant)	79.027	4.065	19.44	0	82.083	4.139	19.83	0	80.786	4.563	17.706	0	87.213	9.652	9.036	<0.001	84.984	11.671	7.282	<0.001
Age (years)	−0.071	0.07	−1.014	0.311	0	0.072	−0.005	0.996	0.006	0.072	0.086	0.932	0.006	0.073	0.077	0.938	−0.003	0.077	−0.038	0.97
Gender	−1.151	1.758	−0.655	0.513	−0.897	1.74	−0.516	0.606	−0.882	1.801	−0.49	0.624	−0.557	1.904	−0.293	0.77	−0.397	1.935	−0.205	0.837
# TBI's	−0.591	0.415	−1.423	0.155	−0.477	0.41	−1.164	0.244	−0.525	0.412	−1.273	0.203	−0.504	0.427	−1.181	0.238	−0.421	0.442	−0.952	0.341
TMT A					−0.097	0.081	−1.196	0.232	−0.099	0.082	−1.197	0.231	−0.13	0.093	−1.39	0.168	−0.15	0.092	−1.629	0.105
TMT B					−0.051	0.027	−1.892	0.058	−0.044	0.028	−1.593	0.111	−0.041	0.034	−1.185	0.241	−0.048	0.043	−1.097	0.287
Anti-saccade latency									28.605	14.657	1.952	0.051	32.044	15.498	2.068	**0.039**	30.762	17.216	1.787	0.078
Anti-saccade error									−11.834	8.399	−1.409	0.159	−12.415	9.474	−1.31	0.193	−13.373	9.264	−1.444	0.15
Anti-saccade accuracy									4.453	3.09	1.441	0.15	4.827	3.098	1.558	0.119	4.789	3.351	1.429	0.155
Distractor P300 amplitude PZ													−0.287	0.34	−0.845	0.416	−0.198	0.431	−0.46	0.657
Distractor P300 latency PZ													0.004	0.011	0.339	0.738	−0.002	0.013	−0.128	0.899
Distractor P300 amplitude FZ													−0.094	0.202	−0.466	0.645	−0.076	0.254	−0.299	0.768
Distractor P300 latency FZ													−0.004	0.009	−0.428	0.67	−0.011	0.012	−0.879	0.388
Distractor N200 amplitude PZ													−0.013	0.339	−0.039	0.97	−0.074	0.451	−0.164	0.872
Distractor N200 latency PZ													0.006	0.025	0.248	0.809	0.005	0.031	0.148	0.886
Distractor N200 amplitude FZ													−0.019	0.248	−0.076	0.941	−0.102	0.322	−0.318	0.757
Distractor N200 latency FZ													−0.026	0.021	−1.242	0.23	−0.033	0.024	−1.368	0.187
Target P300 amplitude PZ																	−0.343	0.368	−0.933	0.372
Target P300 latency PZ																	0.01	0.022	0.459	0.662
Target P300 amplitude FZ																	0.055	0.235	0.235	0.816
Target P300 latency FZ																	0.01	0.012	0.832	0.41
Target N200 amplitude PZ																	0.159	0.377	0.421	0.678
Target N200 latency PZ																	0.007	0.02	0.331	0.742
Target N200 amplitude FZ																	0.043	0.263	0.164	0.871
Target N200 latency FZ																	0.014	0.025	0.56	0.584

B being the coefficient, t is the coefficient divided by its standard error, and the p-value represents the significance level.

### 3.3. SOT1: Eyes open with fixed surface and visual surroundings

Table 3 presents the complete hierarchical regression results for the SOT1 score, the balance score when all sensory information was available and reliable. Step 1 accounted for 5.6% of the variance; none of the demographic measures showed sufficient evidence of an association with SOT1. Step 2 added TMT scores to the model, increased the variance accounted for 9.1%, and TMT Part B was negatively associated with SOT1 (*p* = 0.038), meaning longer times on the TMT-B reflected worse SOT1 scores. Slower time on TMT was associated with increased sway on SOT1. Step 3 added anti-saccade measures, increased the variance accounted for 11.2%, and showed insufficient evidence of any associations. Step 4 added distractor EEG measures and increased variance accounted for 17.3%, but none of the measures were statistically significant in the model. Step 5 added EEG target measures and increased variance accounted for 19.9%. None of the measures showed sufficient evidence of a significant association with SOT1.

**Table 3:** Results from the hierarchical regression for SOT1, standing balance.

**SOT1**	**Model 1**				**Model 2**				**Model 3**				**Model 4**				**Model 5**			
	***B***	**SE**	***t***	***p*-Value**	***B***	**SE**	***t***	***p*-Value**	***B***	**SE**	***t***	***p*-Value**	***B***	**SE**	***t***	***p*-Value**	***B***	**SE**	***t***	***p*-Value**
*R* ^2^	0.056				0.091				0.112				0.173				0.199			
(Constant)	91.031	2.185	41.666	0	92.033	2.291	40.17	0	90.249	2.601	34.699	0	95.599	5.509	17.354	<0.001	94.065	5.774	16.292	<0.001
Age (years)	0.027	0.036	0.765	0.446	0.045	0.037	1.225	0.222	0.043	0.036	1.183	0.238	0.035	0.041	0.865	0.393	0.039	0.044	0.896	0.378
Gender	−0.135	0.987	−0.137	0.892	−0.172	0.961	−0.179	0.859	0.203	1.021	0.199	0.844	0.17	1	0.17	0.866	0.305	0.982	0.311	0.757
# TBI's	−0.321	0.219	−1.469	0.145	−0.277	0.216	−1.28	0.204	−0.254	0.213	−1.191	0.236	−0.266	0.235	−1.129	0.266	−0.268	0.23	−1.164	0.25
TMT A					0.008	0.049	0.17	0.866	−0.008	0.05	−0.153	0.88	−0.013	0.046	−0.291	0.772	−0.026	0.051	−0.518	0.609
TMT B					−0.028	0.014	−2.081	**0.038**	−0.023	0.014	−1.607	0.11	−0.019	0.017	−1.129	0.269	−0.02	0.016	−1.287	0.2
Anti-saccade latency									8.405	7.385	1.138	0.257	11.511	8.472	1.359	0.184	10.306	7.923	1.301	0.197
Anti-saccade error									2.395	4.567	0.524	0.602	0.352	4.539	0.077	0.939	0.459	4.381	0.105	0.917
Anti-saccade accuracy									0.568	1.536	0.37	0.712	0.994	1.639	0.607	0.547	0.775	1.567	0.494	0.622
Distractor P300 amplitude PZ													−0.133	0.191	−0.695	0.509	−0.183	0.155	−1.176	0.255
Distractor P300 latency PZ													−0.002	0.006	−0.273	0.79	−0.005	0.007	−0.649	0.53
Distractor P300 amplitude FZ													−0.024	0.099	−0.244	0.81	0.017	0.118	0.144	0.887
Distractor P300 latency FZ													0	0.006	−0.029	0.978	−9.70E−05	0.009	−0.01	0.992
Distractor N200 amplitude PZ													−0.116	0.198	−0.587	0.572	−0.083	0.195	−0.426	0.676
Distractor N200 latency PZ													0.008	0.016	0.474	0.652	0	0.014	−0.028	0.978
Distractor N200 amplitude FZ													0.114	0.11	1.039	0.316	0.093	0.112	0.827	0.415
Distractor N200 latency FZ													−0.022	0.011	−1.966	0.076	−0.02	0.012	−1.72	0.104
Target P300 amplitude PZ																	0.008	0.121	0.07	0.945
Target P300 latency PZ																	0.007	0.01	0.681	0.52
Target P300 amplitude FZ																	−0.035	0.121	−0.285	0.779
Target P300 latency FZ																	−0.002	0.006	−0.26	0.798
Target N200 amplitude PZ																	0.011	0.139	0.082	0.935
Target N200 latency PZ																	0.014	0.013	1.078	0.303
Target N200 amplitude FZ																	0.042	0.1	0.426	0.671
Target N200 latency FZ																	−0.002	0.016	−0.102	0.922

B being the coefficient, t is the coefficient divided by its standard error, and the p-value represents the significance level.

### 3.4. SOT2: Eyes closed with fixed surface

Table 4 presents the complete hierarchical regression results for the SOT2, balance with eyes closed. Step 1 accounted for 8.9% of the variance. The number of mTBIs showed a negative association with the SOT2 score, indicating participants with higher numbers of lifetime mTBIs had a lower SOT2 score (*p* = 0.002), indicating greater sway. Step 2 added TMT scores to the model and increased the variance accounted for 10.5%, yet none of the TMT measures showed sufficient evidence of an association standing with eyes closed. Step 3 added anti-saccade measures, increased the variance accounted for 11.5%, and showed insufficient evidence of an association with sway during standing with eyes closed. Step 4 added distractor EEG measures and decreased variance accounted for 18.4%, but none of the measures were statistically significant in the model. Step 5 added EEG target measures and increased variance accounted for 20.7%. None of the EEG measures showed sufficient evidence of an association with the SOT2 score.

**Table 4:** Results from the hierarchical regression for SOT2, occluded vision.

**SOT2**	**Model 1**				**Model 2**				**Model 3**				**Model 4**				**Model 5**			
	***B***	**SE**	***t***	***p*-Value**	***B***	**SE**	***t***	***p*-Value**	***B***	**SE**	***t***	***p*-Value**	***B***	**SE**	***t***	***p*-Value**	***B***	**SE**	***t***	***p*-Value**
*R* ^2^	0.089				0.105				0.115				0.184				0.207			
(Constant)	89.497	2.947	30.371	0	90.791	2.963	30.643	0	89.537	3.263	27.436	0	91.699	8.079	11.351	<0.001	88.646	9.591	9.242	<0.001
Age (years)	−0.011	0.05	−0.212	0.832	0.02	0.054	0.381	0.704	0.025	0.055	0.465	0.643	0.007	0.06	0.115	0.909	0.002	0.065	0.03	0.976
Gender	0.065	1.345	0.048	0.962	0.198	1.388	0.142	0.887	0.445	1.483	0.3	0.765	0.77	1.301	0.592	0.554	0.944	1.341	0.704	0.482
# TBI's	−0.93	0.293	−3.171	**0.002**	−0.883	0.293	−3.01	**0.003**	−0.865	0.293	−2.954	**0.003**	−0.815	0.33	−2.472	**0.016**	−0.751	0.337	−2.229	**0.03**
TMT A					−0.05	0.063	−0.791	0.431	−0.051	0.064	−0.807	0.422	−0.057	0.071	−0.806	0.426	−0.075	0.07	−1.075	0.289
TMT B					−0.019	0.02	−0.966	0.334	−0.015	0.02	−0.757	0.45	−0.017	0.023	−0.759	0.451	−0.02	0.029	−0.689	0.499
Anti-saccade latency									10.062	12.021	0.837	0.407	13.858	12.178	1.138	0.262	13.621	12.684	1.074	0.29
Anti-saccade error									−4.575	6.078	−0.753	0.452	−6.089	6.273	−0.971	0.333	−6.524	6.282	−1.039	0.3
Anti-saccade accuracy									0.278	2.737	0.101	0.92	0.468	2.487	0.188	0.852	0.379	2.6	0.146	0.885
Distractor P300 amplitude PZ													−0.153	0.17	−0.901	0.372	−0.141	0.219	−0.646	0.524
Distractor P300 latency PZ													0.002	0.01	0.202	0.845	−0.003	0.007	−0.425	0.671
Distractor P300 amplitude FZ													−0.114	0.12	−0.953	0.342	−0.098	0.151	−0.646	0.521
Distractor P300 latency FZ													−0.004	0.009	−0.422	0.682	−0.007	0.01	−0.718	0.487
Distractor N200 amplitude PZ													−0.225	0.319	−0.703	0.503	−0.273	0.422	−0.647	0.539
Distractor N200 latency PZ													0.025	0.022	1.15	0.289	0.022	0.024	0.913	0.39
Distractor N200 amplitude FZ													−0.032	0.187	−0.172	0.867	−0.041	0.207	−0.196	0.848
Distractor N200 latency FZ													−0.027	0.018	−1.515	0.164	−0.033	0.018	−1.786	0.097
Target P300 amplitude PZ																	−0.129	0.19	−0.676	0.503
Target P300 latency PZ																	0.011	0.017	0.658	0.536
Target P300 amplitude FZ																	0.03	0.158	0.19	0.851
Target P300 latency FZ																	0.006	0.008	0.74	0.462
Target N200 amplitude PZ																	0.166	0.304	0.545	0.596
Target N200 latency PZ																	0.009	0.02	0.466	0.652
Target N200 amplitude FZ																	−0.069	0.182	−0.377	0.71
Target N200 latency FZ																	0.005	0.021	0.235	0.82

B being the coefficient, t is the coefficient divided by its standard error, and the p-value represents the significance level.

### 3.5. SOT3: Eyes open with fixed surface and sway-referenced visual surroundings

Table 5 presents the complete hierarchical regression results for the SOT3, the balance score when standing with a sway reference surround. Step 1 accounted for 5.3% of the variance, but none of the demographic measures showed evidence of an association with SOT3. Step 2 added TMT scores to the model, increased the variance accounted for 10.9%, and revealed that none of the TMT measures showed sufficient evidence of an association with the SOT 3. Step 3 added anti-saccade measures, increased the variance accounted for 11.7%, and showed insufficient evidence of an association with the SOT3. Step 4 added distractor EEG measures and increased variance accounted for 18.3%, but none of the measures were statistically significant in the model. Step 5 added EEG target measures and increased variance accounted for 21.5%. The distractor N200 latency at the FZ location showed a negative association with the SOT3 score (*p* = 0.019).

**Table 5:** Results from the hierarchical regression for SOT3, sway-referenced vision.

**SOT3**	**Model 1**				**Model 2**				**Model 3**				**Model 4**				**Model 5**			
	***B***	**SE**	***t***	***p*-Value**	***B***	**SE**	***t***	***p*-Value**	***B***	**SE**	***t***	***p*-Value**	***B***	**SE**	***t***	***p*-Value**	***B***	**SE**	***t***	***p*-Value**
*R* ^2^	0.053				0.109				0.117				0.183				0.215			
(Constant)	88.701	3.798	23.353	0	91.111	4.019	22.668	0	90.646	4.433	20.45	<0.001	96.414	8.962	10.759	<0.001	94.294	13.038	7.232	<0.001
Age (years)	−7.70E−06	0.065	0	1	0.057	0.061	0.939	0.35	0.065	0.061	1.055	0.294	0.056	0.066	0.852	0.4	0.053	0.071	0.748	0.462
Gender	−0.273	1.519	−0.18	0.858	−0.034	1.499	−0.022	0.982	0.019	1.607	0.012	0.991	0.18	1.501	0.12	0.905	0.399	1.403	0.284	0.776
# TBI's	−0.572	0.317	−1.802	0.072	−0.484	0.313	−1.547	0.122	−0.482	0.319	−1.512	0.131	−0.419	0.325	−1.289	0.198	−0.373	0.336	−1.109	0.269
TMT A					−0.09	0.086	−1.053	0.308	−0.082	0.085	−0.971	0.344	−0.102	0.09	−1.131	0.276	−0.115	0.081	−1.43	0.165
TMT B					−0.037	0.022	−1.634	0.104	−0.035	0.023	−1.506	0.134	−0.03	0.028	−1.058	0.301	−0.026	0.036	−0.733	0.478
Anti-saccade latency									9.233	11.486	0.804	0.422	13.44	13.637	0.986	0.331	14.86	13.264	1.12	0.268
Anti-saccade error									−7.842	6.827	−1.149	0.252	−8.127	7.71	−1.054	0.299	−8.42	7.736	−1.088	0.284
Anti-saccade accuracy									0.439	2.645	0.166	0.869	0.856	2.866	0.299	0.767	0.825	2.875	0.287	0.776
Distractor P300 amplitude PZ													−0.158	0.213	−0.744	0.466	−0.196	0.233	−0.839	0.409
Distractor P300 latency PZ													0.005	0.012	0.417	0.689	0.003	0.008	0.333	0.739
Distractor P300 amplitude FZ													−0.112	0.158	−0.709	0.486	−0.162	0.201	−0.804	0.433
Distractor P300 latency FZ													−0.008	0.009	−0.874	0.401	−0.012	0.011	−1.136	0.28
Distractor N200 amplitude PZ													−0.088	0.309	−0.286	0.781	−0.089	0.458	−0.195	0.852
Distractor N200 latency PZ													0.015	0.021	0.714	0.496	0.014	0.024	0.578	0.578
Distractor N200 amplitude FZ													−0.095	0.175	−0.543	0.595	−0.059	0.283	−0.207	0.842
Distractor N200 latency FZ													−0.035	0.022	−1.606	0.149	−0.044	0.017	−2.529	**0.019**
Target P300 amplitude PZ																	−0.062	0.175	−0.355	0.723
Target P300 latency PZ																	0.003	0.022	0.134	0.899
Target P300 amplitude FZ																	0.113	0.171	0.657	0.517
Target P300 latency FZ																	0.009	0.011	0.81	0.434
Target N200 amplitude PZ																	0.023	0.34	0.066	0.948
Target N200 latency PZ																	0.01	0.027	0.363	0.727
Target N200 amplitude FZ																	−0.092	0.25	−0.368	0.721
Target N200 latency FZ																	0.003	0.019	0.146	0.886

B being the coefficient, t is the coefficient divided by its standard error, and the p-value represents the significance level.

### 3.6. SOT4: Eyes open with sway-referenced surface and fixed visual surroundings

Step 1 accounted for 2.0% of the variance of the SOT4 score. None of the demographic measures showed evidence of an association with the SOT4. Step 2 added TMT scores to the model, increased the variance accounted for 9.7%, and revealed that none of the TMT measures showed evidence of an association with the SOT4 score. Step 3 added anti-saccade measures, increased the variance accounted for 10.6%, and showed no evidence of an association with the SOT4. Step 4 added distractor EEG measures and increased variance accounted for 14.5%, but none of the measures were significant in the model. Step 5 added EEG target measures and increased variance accounted for 16.9%. None of the measures showed evidence of an association with the SOT4 score. Refer to Supplementary material for complete results.

### 3.7. SOT5: Eyes closed with a sway-referenced surface

Table 6 presents the complete hierarchical regression results for the SOT5 score, the balance score with eyes closed, and a sway-referenced surface. Step 1 accounted for 4.6% of the variance of the SOT5 score. None of the demographic measures showed sufficient evidence of an association with the SOT5. Step 2 added TMT scores to the model, increased the variance accounted for 6.6%, and revealed that none of the TMT measures showed sufficient evidence of an association with the SOT5 score. Step 3 added anti-saccade measures, increased the variance accounted for 9.5%, and showed an association of anti-saccade latency and error (*p* = 0.026 and *p* = 0.043, respectively) with the SOT5, indicating longer latencies and less error were associated with better balance scores. Step 4 added distractor EEG measures and increased variance accounted for 13.2%, but only anti-saccade latency was statistically significant in the model. Step 5 added EEG target measures and increased variance accounted for 16.4%. None of the measures showed sufficient evidence of an association with the SOT5 score.

**Table 6:** Results from the hierarchical regression for SOT5, eyes closed with a sway-referenced surface.

**SOT5**	**Model 1**				**Model 2**				**Model 3**				**Model 4**				**Model 5**			
	***B***	**SE**	***t***	***p*-Value**	***B***	**SE**	***t***	***p*-Value**	***B***	**SE**	***t***	***p*-Value**	***B***	**SE**	***t***	***p*-Value**	***B***	**SE**	***t***	***p*-Value**
*R* ^2^	0.046				0.066				0.095				0.132				0.164			
(Constant)	68.231	5.909	11.548	0	70.539	6.054	11.651	0	69.151	6.519	10.607	0	72.891	13.013	5.602	<0.001	69.475	13.889	5.002	<0.001
Age (years)	−0.103	0.101	−1.026	0.305	−0.056	0.106	−0.529	0.597	−0.041	0.107	−0.382	0.703	−0.037	0.118	−0.311	0.756	−0.047	0.121	−0.385	0.701
Gender	−1.597	2.618	−0.61	0.542	−1.541	2.647	−0.582	0.561	−1.716	2.708	−0.634	0.526	−1.202	3.034	−0.396	0.693	−0.952	3.068	−0.31	0.757
# TBI's	−0.775	0.607	−1.277	0.202	−0.681	0.608	−1.119	0.263	−0.778	0.612	−1.271	0.204	−0.675	0.636	−1.061	0.289	−0.514	0.633	−0.812	0.417
TMT A					−0.028	0.12	−0.235	0.814	−0.017	0.122	−0.142	0.887	−0.072	0.137	−0.522	0.603	−0.097	0.131	−0.745	0.456
TMT B					−0.052	0.041	−1.261	0.207	−0.042	0.042	−0.993	0.321	−0.032	0.049	−0.642	0.523	−0.05	0.062	−0.807	0.429
Anti-saccade latency									48.66	21.854	2.227	**0.026**	50.612	22.872	2.213	**0.027**	49.037	24.742	1.982	**0.05**
Anti-saccade error									−25.364	12.517	−2.026	**0.043**	−24.163	13.179	−1.833	0.067	−25.794	12.872	−2.004	**0.045**
Anti-saccade accuracy									7.588	4.602	1.649	0.1	7.431	4.578	1.623	0.105	7.756	4.962	1.563	0.12
Distractor P300 amplitude PZ													−0.261	0.564	−0.462	0.656	−0.113	0.648	−0.174	0.866
Distractor P300 latency PZ													0.005	0.023	0.208	0.841	−0.005	0.026	−0.189	0.854
Distractor P300 amplitude FZ													−0.12	0.359	−0.334	0.744	−0.149	0.487	−0.305	0.767
Distractor P300 latency FZ													0.001	0.018	0.05	0.961	−0.009	0.02	−0.44	0.667
Distractor N200 amplitude PZ													0.202	0.47	0.429	0.671	0	0.61	0.001	1
Distractor N200 latency PZ													−0.002	0.028	−0.06	0.952	0.001	0.045	0.024	0.981
Distractor N200 amplitude FZ													−0.215	0.352	−0.611	0.551	−0.324	0.464	−0.697	0.501
Distractor N200 latency FZ													−0.019	0.033	−0.576	0.573	−0.033	0.042	−0.785	0.449
Target P300 amplitude PZ																	−0.561	0.527	−1.065	0.309
Target P300 latency PZ																	0.017	0.025	0.67	0.519
Target P300 amplitude FZ																	0.206	0.442	0.467	0.651
Target P300 latency FZ																	0.013	0.02	0.642	0.528
Target N200 amplitude PZ																	0.436	0.585	0.747	0.464
Target N200 latency PZ																	−9.00E−05	0.04	−0.002	0.998
Target N200 amplitude FZ																	−0.026	0.47	−0.056	0.956
Target N200 latency FZ																	0.03	0.041	0.731	0.481

B being the coefficient, t is the coefficient divided by its standard error, and the p-value represents the significance level.

### 3.8. SOT6: Eyes open with sway-referenced surface and visual surroundings

Step 1 accounted for 5.3% of the variance of the SOT6 score. None of the demographic measures showed sufficient evidence of an association with the SOT6. Step 2 added TMT scores to the model, increased the variance accounted for 8.8%, and revealed that none of the TMT measures showed sufficient evidence of an association with the SOT6 score. Step 3 added anti-saccade measures, increased the variance accounted for 10.5%, and showed insufficient evidence of an association with the SOT6. Step 4 added distractor EEG measures and increased variance accounted for 12.8%, but none of the measures were statistically significant in the model. Step 5 added EEG target measures and increased variance accounted for 15.2%. None of the measures showed sufficient evidence of an association with the SOT6 score. Refer to Supplementary material for complete results.

## 4. Discussion

The goal of this study was to determine the relationships between EF and postural balance among current and former combat-exposed service members, with and without a history of mTBI(s). Maintaining balance requires combining and processing sensory information while adjusting the weighting of this sensory information based on the fidelity of these signals (15). Thus, the fidelity of the sensory information, as well as the ability to process and weigh this information, affects balance. This study reinforces that postural balance is a complex control problem that utilizes multiple sensory systems and requires the ability to successfully process multiple inputs at the executive processing level.

In general, individuals with past mTBI(s) can maintain postural balance (SOT 1, 3, 4, 5, 6, and composite) and successfully ambulate. However, individuals with mTBI have more difficulty with adjustments when standing with their eyes closed (SOT 2). In prior research, Haran et al. (35) found that service members with an mTBI scored worse on the SOT-composite score, mostly due to worse scores on the SOT4 and SOT5, a sway-referenced standing surface with, respectively, eyes open and occluded. However, they did not find a significant difference between service members with a positive or negative history of mTBI and the SOT2. This difference in findings could be due to a large number of service members with an acute TBI (within 5 days of TBI) in their study. In contrast, participants in our study were in the chronic phase (median 9 years since the last mTBI). Additionally, all of their cohorts had blast-related mTBI, which may have different pathophysiology from non-blast mTBI, whereas our cohort included both types.

In general, few associations were found relating EF to balance measures. All EF measures combined (demographics, TMT, anti-saccade, and oddball assessment) accounted for between 15.2% and 21.5% of the variability in the balance measures. While this may seem limited, these models do not account for many factors that affect gait and balance. For example, physical activity levels (36, 37) and hand grip strength (38), known to be associated with balance and gait measures, are not accounted for in this study.

Nevertheless, we found that a longer time to complete TMT Part B was related to worse standing balance (SOT1). The TMT Part B reflects executive functioning, visual scanning, short-term working memory, and complex attention (39, 40). Earlier research found relationships between lower TMT scores and worse scores on lower limb function tests (including timed up and go, 4-m walk standing balance tests) (41, 42), with the TMT being predictive of a worse short physical performance battery score (4-m walk test, five-time rise from chair without using hands, standing balance with feet side by side, semi-tandem, and tandem) based on a 6-year follow-up (42). Together these results support the importance of EF for the integration of sensory information to maintain balance.

Longer anti-saccade response time was associated with a better SOT-composite score and better balance score with a sway-referenced surface and eyes closed (SOT5). Better SOT5 scores were also associated with less error on anti-saccade initial directionality. This indicates that when one can only rely on vestibular information, a faster response may not always be better but taking more time to process information to react more accurately benefits balance. This is counter-intuitive as long delays are associated with later actions to maintain balanced. However, this long latency may indicate the inhibition of the impulse to saccade to the target and directly make a correct anti-saccade away from the target, especially considering the association with an accuracy of the amplitude of the anti-saccade and the SOT5 as well, and the fact that in the SOT5, the time has to be taken to inhibit proprioception without the use of vision and only rely on the vestibular system.

Electroencephalogram distractor measures at FZ (N200 minimum latency) were associated with balance measures when proprioception and vestibular were reliable sensory inputs, but the vision was sway-referenced (SOT3). This indicates that greater sway (and poorer ability to suppress inaccurate visual information from the sway-referenced scene) is related to longer conflict monitoring time as reflected in the N200 latency during auditory distraction). This may indicate that people who have more difficulty inhibiting task-irrelevant information or resolving competing responses also have difficulty directing away from vision to other modalities to maintain balance.

Patients with mTBI are known to present with both high rates of vestibular and proprioception impairments and dysfunction (35, 43–45). This high rate of impairments in vestibular and proprioception function could explain the lack of associations found between EF measures and the balance tests that feed misleading proprioception cues (a sway reference standing surface in SOT4, 5, and 6) in the mTBI population.

### 4.1. Limitations

It is worth noting that 53.85% of non-mTBI participants had relatively low-SOT-composite scores (<75). Based on the manufacturer's stated normative data with mean (SD) composite scores equal to 79.8 (5.7), we would expect only 20% of “normal” individuals to have composite scores below 75. The higher proportion in our sample may be due to comorbidities, including chronic pain, PTSD, and sleep apnea in veterans and service members (46), which previous preliminary analyses have linked to lower SOT-composite scores in Veterans and Service Members (47) than the general population. In the future, similar analyses may be performed with a control group more representative of the general public. Given that our sample had all served in the military and was predominantly male, results may not generalize to civilian or female populations.

Additionally, a large proportion of the participants had missing data; however, we imputed the missing values known to reduce bias and improve efficiency over complete case analysis (48, 49). The highest rate of missing data was for EEG (over 55%), so the results of EEG distractor measures are considered very tentative and may only be useful for formulating future hypotheses (50, 51).

## 5. Conclusion

In agreement with earlier research showing that EF measures taken during balance are altered in people with compromised balance, we found some associations between EF and postural stability on several sensory conditions of the SOT test. This included N200 amplitude reduction and P300 latency delay when the ability to depend on vestibular or proprioception is taxed by corrupting visual information (SOT3) or longer response latencies in an inhibition task when only reliable vestibular information is available (SOT5). In addition, the lack of associations with EF measures and the other SOT tests that corrupt proprioception could indicate that the combat-exposed cohort has multifactorial vestibular and proprioception deficits, making them more dependent on their visual information for the maintenance of balance.

## Data availability statement

The data analyzed in this study is subject to the following licenses/restrictions. The CENC/LIMBIC data board has to approve data availability. Requests to access these datasets should be directed at: Sudeep Karki Sudeep Karki@vcuhealth.org.

## Ethics statement

The studies involving human participants were reviewed and approved by Richmond VAMC Research and Development committee. The patients/participants provided their written informed consent to participate in this study.

## Author contributions

SV contributed to conceptualization, analysis, and writing of the first draft. RP contributed to conceptualization, analysis, statistical advice, and proofreading of the manuscript. LM-F contributed to EEG data analysis and proofreading of the manuscript. AA, KS, SS, EW, AS, JT, and WW contributed to conceptualizion, analysis methods, and proofreading of the manuscript. All authors contributed to the article and approved the submitted version.

## References

[1] Roerdink M Lamoth CJC van Kordelaar J Elich P Konijnenbelt M Kwakkel G. Rhythm perturbations in acoustically paced treadmill walking after stroke. Neurorehabil Neural Repair. (2009) 23:668–78. 10.1177/1545968309332879 19307435

[2] van der Veen SM Hammerbeck U Hollands KL. Foot-placement accuracy during planned and reactive target stepping during walking in stroke survivors and healthy adults. Gait Posture. (2020) 81:261–7. 10.1016/j.gaitpost.2020.08.114 32846357

[3] Mazaheri M Hoogkamer W Potocanac Z Verschueren S Roerdink M Beek PJ. Effects of aging and dual tasking on step adjustments to perturbations in visually cued walking. Exp Brain Res. (2015) 233:3467–74. 10.1007/s00221-015-4407-5 26298043 PMC4646946

[4] Lundin-Olsson L Nyberg L Gustafson Y. Stops walking when talking” as a predictor of falls in elderly people. Lancet. (1997) 349:617. 10.1016/S0140-6736(97)24009-2 9057736

[5] Oh C Morris RJ LaPointe LL Stierwalt JAG. Spatial-temporal parameters of gait associated with alzheimer disease: a longitudinal analysis. J Geriatr Psychiatry Neurol. (2021) 34:46–59. 10.1177/0891988720901779 32129132

[6] Coonley-Hoganson R Sachs N Desai BT Whitman S. Sequelae associated with head injuries in patients who were not hospitalized: a follow-up survey. Neurosurgery. (1984) 14:315–7. 10.1227/00006123-198403000-00009 6709157

[7] Bazarian JJ Wong T Harris M Leahey N Mookerjee S Dombovy M. Epidemiology and predictors of post-concussive syndrome after minor head injury in an emergency population. Brain Injury. (1999) 13:173–89. 10.1080/026990599121692 10081599

[8] Middleboe T Andersen HS Birket-Smith M Friis ML. Minor head injury: impact on general health after 1 year. A prospective follow-up study. Acta Neurol Scand. (2009) 85:5–9. 10.1111/j.1600-0404.1992.tb03987.x 1546534

[9] Hoge CW McGurk D Thomas JL Cox AL Engel CC Castro CA. Mild traumatic brain injury in US soldiers returning from Iraq. N Engl J Med. (2008) 358:453–63. 10.1056/NEJMoa072972 18234750

[10] Pogoda TK Hendricks AM Iverson KM Stolzmann KL Krengel MH Baker E. Multisensory impairment reported by veterans with and without mild traumatic brain injury history. J Rehabil Res Dev. (2012) 49:971–84. 10.1682/JRRD.2011.06.0099 23341273

[11] Terrio H Brenner LA Ivins BJ Cho JM Helmick K Schwab K. Traumatic brain injury screening: preliminary findings in a US Army Brigade Combat Team. J Head Trauma Rehabil. (2009) 24:14–23. 10.1097/HTR.0b013e31819581d8 19158592

[12] Vallée M McFadyen BJ Swaine B Doyon J Cantin J-F Dumas D. Effects of environmental demands on locomotion after traumatic brain injury. Arch Phys Med Rehabil. (2006) 87:806–13. 10.1016/j.apmr.2006.02.031 16731216

[13] van Donkelaar P Osternig L Chou LS. Attentional and biomechanical deficits interact after mild traumatic brain injury. Exerc Sport Sci Rev. (2006) 34:77–82. 10.1249/00003677-200604000-00007 16672805

[14] McCulloch K. Attention and dual-task conditions: physical therapy implications for individuals with acquired brain injury. J Neurol Phys Ther. (2007) 31:104–18. 10.1097/NPT.0b013e31814a6493 18025956

[15] Peterka RJ. Sensorimotor integration in human postural control. J Neurophysiol. (2002) 88:1097–118. 10.1152/jn.2002.88.3.1097 12205132

[16] Dichter GS van der Stelt O Boch JL Belger A. Relations among intelligence, executive function, and P300 event related potentials in schizophrenia. J Nerv Ment Dis. (2006) 194:179–87. 10.1097/01.nmd.0000202490.97425.de 16534435

[17] Huster RJ Enriquez-Geppert S Lavallee CF Falkenstein M Herrmann CS. Electroencephalography of response inhibition tasks: functional networks and cognitive contributions. Int J Psychophysiol. (2013) 87:217–33. 10.1016/j.ijpsycho.2012.08.001 22906815

[18] Bradford JC Lukos JR Passaro A Ries A Ferris DP. Effect of locomotor demands on cognitive processing. Sci Rep. (2019) 9:9234. 10.1038/s41598-019-45396-5 31239461 PMC6592922

[19] Nordin AD Hairston WD Ferris DP. Faster gait speeds reduce alpha and beta EEG spectral power from human sensorimotor cortex. IEEE Trans Biomed Eng. (2019) 67:842–53. 10.1109/TBME.2019.2921766 31199248 PMC7134343

[20] Peterson SM Ferris DP. Differentiation in theta and beta electrocortical activity between visual and physical perturbations to walking and standing balance. eNeuro. (2018). 5:ENEURO.0207-18.2018. 10.1523/ENEURO.0207-18.2018 30105299 PMC6088363

[21] Nordin AD Hairston WD Ferris DP. Human electrocortical dynamics while stepping over obstacles. Sci Rep. (2019) 9:4693. 10.1038/s41598-019-41131-2 30886202 PMC6423113

[22] Lord S Galna B Verghese J Coleman S Burn D Rochester L. Independent domains of gait in older adults and associated motor and nonmotor attributes: validation of a factor analysis approach. J Gerontol A Biol Sci Med Sci. (2013) 68:820–7. 10.1093/gerona/gls255 23250001

[23] Morris R Martini DN Smulders K Kelly VE Zabetian CP Poston K. Cognitive associations with comprehensive gait and static balance measures in Parkinson's disease. Parkinsonism Relat Disord. (2019) 69:104–10. 10.1016/j.parkreldis.2019.06.014 31731260 PMC6900452

[24] Walker WC Carne W Franke LM Nolen T Dikmen SD Cifu DX. The Chronic Effects of Neurotrauma Consortium (CENC) multi-centre observational study: description of study and characteristics of early participants. Brain Injury. (2016) 30:1469–80. 10.1080/02699052.2016.1219061 27834538

[25] Corrigan JD Bogner J. Initial reliability and validity of the Ohio State University TBI identification method. J Head Trauma Rehabil. (2007) 22:318–29. 10.1097/01.HTR.0000300227.67748.77 18025964

[26] Walker WC Cifu DX Hudak AM Goldberg G Kunz RD Sima AP. Structured interview for mild traumatic brain injury after military blast: inter-rater agreement and development of diagnostic algorithm. J Neurotrauma. (2015) 32:464–73. 10.1089/neu.2014.3433 25264909

[27] National Center for Chronic Disease Prevention Health Promotion D.o.P.H. Behavioral Risk Factor Surveillance System. (2022). Available online at: https://www.cdc.gov/brfss/ (accessed January 10, 2022).

[28] Cevette MJ Puetz B Marion MS Wertz ML Muenter MD. Aphysiologic performance on dynamic posturography. Otolaryngol Head Neck Surg. (1995) 112:676–88. 10.1016/S0194-59989570175-3 7777351

[29] Ruffolo LF Guilmette TJ Willis GW. Comparison of time and error rates on the trail making test among patients with head injuries, experimental malingerers, patients with suspect effort on testing, and normal controls. Clin Neuropsychol. (2000) 14:223–30. 10.1076/1385-4046(200005)14:2;1-Z;FT223 10916197

[30] Llinàs-Reglà J Vilalta-Franch J López-Pousa S Calvó-Perxas L Torrents-Rodas D Garre-Olmo J. The trail making test: association with other neuropsychological measures and normative values for adults aged 55 years and older from a spanish-speaking population-based sample. Assessment. (2017) 24:183–96. 10.1177/1073191115602552 26318386

[31] Olichney JM Iragui VJ Salmon DP Riggins BR Morris SK Kutas M. Absent event-related potential (ERP) word repetition effects in mild Alzheimer's disease. Clin Neurophysiol. (2006) 117:1319–30. 10.1016/j.clinph.2006.02.022 16644278 PMC1544116

[32] Cifu DX Wares JR Hoke KW Wetzel PA Gitchel G Carne W. Differential eye movements in mild traumatic brain injury versus normal controls. J Head Trauma Rehabil. (2015) 30:21–8. 10.1097/HTR.0000000000000036 24695263

[33] Kramer JH Mungas D Possin KL Rankin KP Boxer AL Rosen HJ. NIH EXAMINER: conceptualization and development of an executive function battery. J Int Neuropsychol Soc. (2014) 20:11–9. 10.1017/S1355617713001094 24103232 PMC4474183

[34] Benjamini Y Hochberg Y. Controlling the false discovery rate: a practical and powerful approach to multiple testing. J R Stat Soc Series B. (1995) 57:289–300. 10.1111/j.2517-6161.1995.tb02031.x

[35] Haran FJ Slaboda JC King LA Wright WG Houlihan D Norris JN. Sensitivity of the balance error scoring system and the sensory organization test in the combat environment. J Neurotrauma. (2016) 33:705–11. 10.1089/neu.2015.4060 26560740

[36] Visser M Pluijm SMF Stel VS Bosscher RJ Deeg DJH Amsterdam LAS. Physical activity as a determinant of change in mobility performance: the Longitudinal Aging Study Amsterdam. J Am Geriatr Soc. (2002) 50:1774–81. 10.1046/j.1532-5415.2002.50504.x 12410894

[37] McMullan II McDonough SM Tully MA Cupples M Casson K Bunting BP. The association between balance and free-living physical activity in an older community-dwelling adult population: a systematic review and meta-analysis. BMC Public Health. (2018) 18:431. 10.1186/s12889-018-5265-4 29609585 PMC5879995

[38] Wiśniowska-Szurlej A Cwirlej-Sozańska A Wołoszyn N Sozański B Wilmowska-Pietruszyńska A. Association between handgrip strength, mobility, leg strength, flexibility, and postural balance in older adults under long-term care facilities. Biomed Res Int. (2019) 2019:1042834. 10.1155/2019/1042834 31662962 PMC6778940

[39] Arbuthnott K Frank J. Trail making test, part B as a measure of executive control: validation using a set-switching paradigm. J Clin Exp Neuropsychol. (2000) 22:518–28. 10.1076/1380-3395(200008)22:4;1-0;FT518 10923061

[40] Kortte KB Horner MD Windham WK. The trail making test, part B: cognitive flexibility or ability to maintain set? Appl Neuropsychol. (2002) 9:106–9. 10.1207/S15324826AN0902_5 12214820

[41] Hirota C Watanabe M Sun W Tanimoto Y Kono R Takasaki K. Association between the Trail Making Test and physical performance in elderly Japanese. Geriatr Gerontol Int. (2010) 10:40–7. 10.1111/j.1447-0594.2009.00557.x 20102381

[42] Vazzana R Bandinelli S Lauretani F Volpato S Lauretani F Di Iorio A. Trail making test predicts physical impairment and mortality in older persons. J Am Geriatr Soc. (2010) 58:719–23. 10.1111/j.1532-5415.2010.02780.x 20398153 PMC2935170

[43] Fino PC Dibble LE Wilde EA Fino NF Johnson P Cortez MM. Sensory Phenotypes for balance dysfunction after mild traumatic brain injury. Neurology. (2022) 99:e521–35. 10.1212/WNL.0000000000200602 35577572 PMC9421603

[44] Fino PC Raffegeau TE Parrington L Peterka RJ King LA. Head stabilization during standing in people with persisting symptoms after mild traumatic brain injury. J Biomech. (2020) 112:110045. 10.1016/j.jbiomech.2020.110045 33011672 PMC8382511

[45] Caccese JB Santos FV Yamaguchi FK Buckley TA Jeka JJ. Persistent visual and vestibular impairments for postural control following concussion: a cross-sectional study in university students. Sports Med. (2021). 10.1007/s40279-021-01472-3 33881749 PMC8449812

[46] Pugh MJV Finley EP Copeland LA Wang C-P Noel PH Amuan ME. Complex comorbidity clusters in OEF/OIF veterans: the polytrauma clinical triad and beyond. Med Care. (2014) 52:172–81. 10.1097/MLR.0000000000000059 24374417

[47] Walker WC Nowak KJ Kenney K Franke LM Eapen BC Skop K. et al. Is balance performance reduced after mild traumatic brain injury?: interim analysis from chronic effects of neurotrauma consortium (CENC) multi-centre study. Brain Injury. (2018) 32:1156–68. 10.1080/02699052.2018.1483529 29894203

[48] Lee KJ Carlin JB. Recovery of information from multiple imputation: a simulation study. Emerg Themes Epidemiol. (2012) 9:3. 10.1186/1742-7622-9-3 22695083 PMC3544721

[49] Madley-Dowd P Hughes R Tilling K Heron J. The proportion of missing data should not be used to guide decisions on multiple imputation. J Clin Epidemiol. (2019) 110:63–73. 10.1016/j.jclinepi.2019.02.016 30878639 PMC6547017

[50] Dong Y Peng CY. Principled missing data methods for researchers. Springerplus. (2013) 2:222. 10.1186/2193-1801-2-222 23853744 PMC3701793

[51] Jakobsen JC Gluud C Wetterslev J Winkel P. When and how should multiple imputation be used for handling missing data in randomised clinical trials - a practical guide with flowcharts. BMC Med Res Methodol. (2017) 17:162. 10.1186/s12874-017-0442-1 29207961 PMC5717805

