# Supplementary figures and images for: Executive function and relation to static balance metrics in chronic mild TBI: A LIMBIC-CENC secondary analysis
Source: Front Neurol. 2023 Jan 11;13:906661. doi: 10.3389/fneur.2022.906661 (PMC9874327; doi:10.3389/fneur.2022.906661)

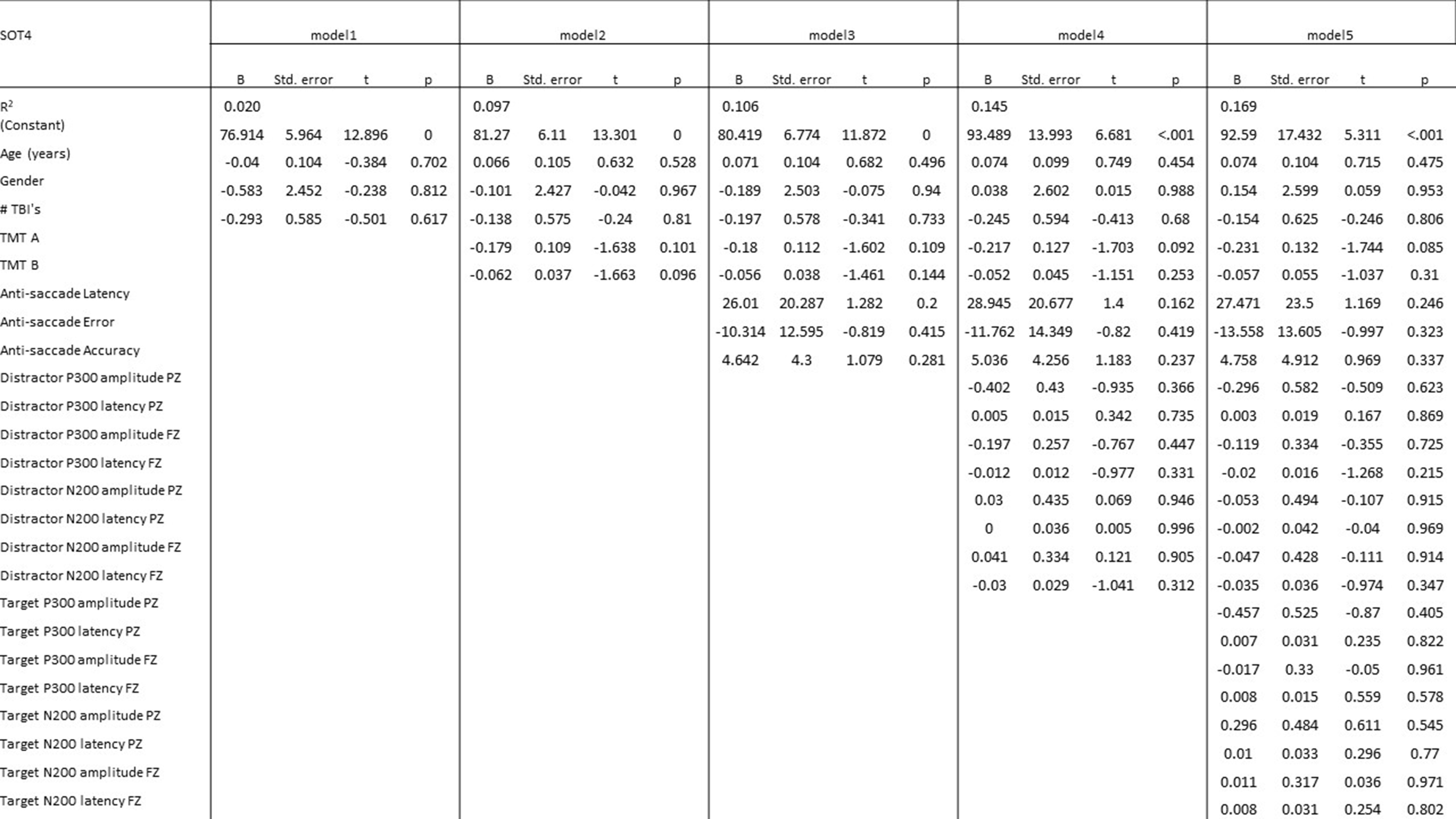

Supplement: Supplementary file 1 [file Image_1.jpeg]

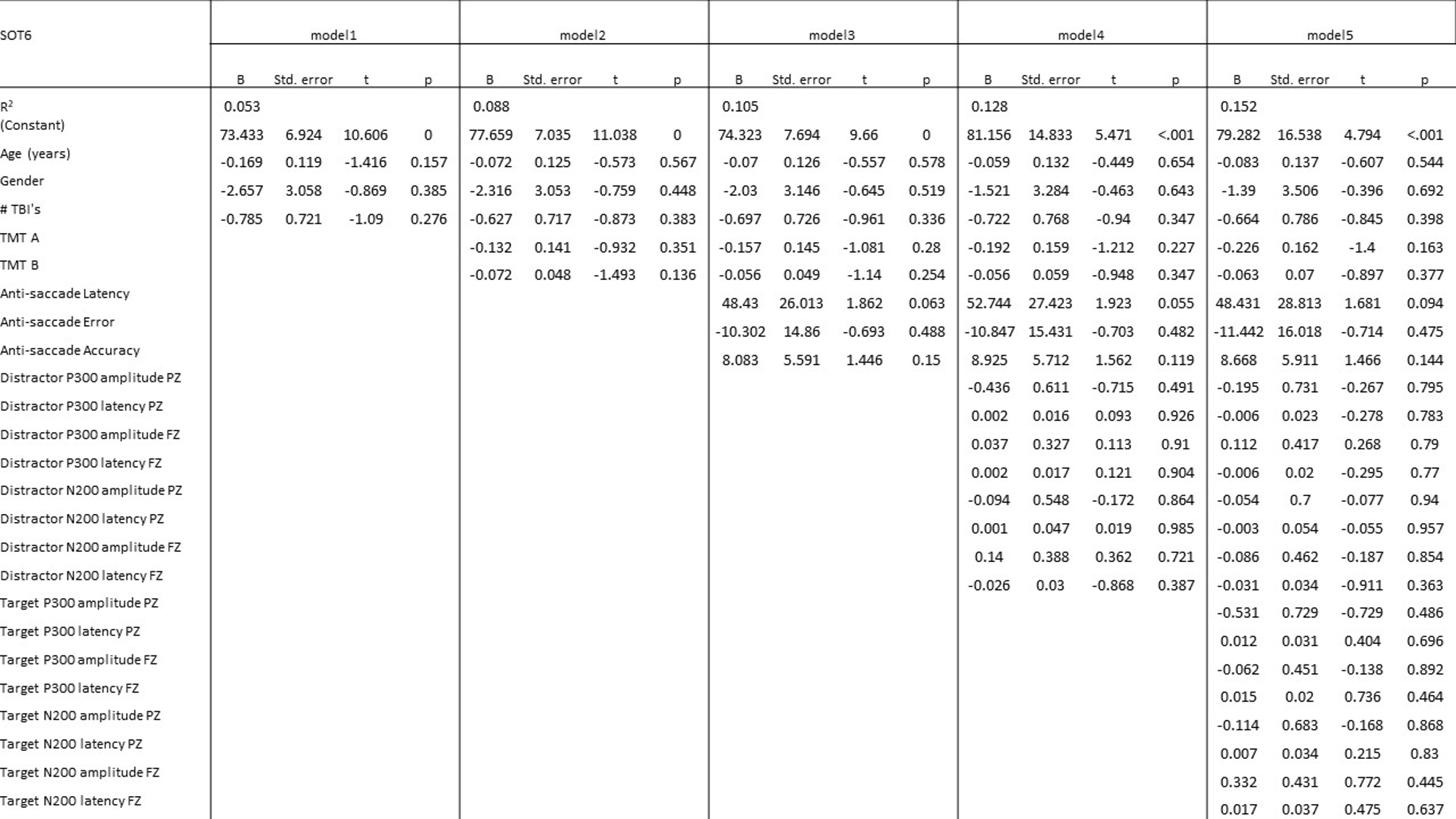

Supplement: Supplementary file 2 [file Image_2.jpeg]
